# Increased deep muscle activity with interference low-frequency electrical muscle stimulation: evaluation by positron emission tomography

**DOI:** 10.1007/s00421-025-05847-6

**Published:** 2025-06-12

**Authors:** Yuichi Nishikawa, Junsuke Nakase, Takuya Sengoku, Seigo Kinuya

**Affiliations:** 1 Faculty of Frontier Engineering, Institute of Science & Engineering, Kanazawa University, Kakuma-machi, Kanazawa, 920-1192 Japan; 2 Department of Orthopaedic Surgery, Graduate School of Medical Science, Kanazawa University, Kanazawa, Japan; 3 Section of Rehabilitation, Kanazawa University Hospital, Kanazawa, Japan; 4 Department of Nuclear Medicine, Faculty of Medicine, Institute of Medical, Pharmaceutical and Health Sciences, Kanazawa University, Kanazawa, Japan

**Keywords:** Electrical muscle stimulation, Interference wave, PET, Muscle activity

## Abstract

**Background:**

The effects of electrical muscle stimulation (EMS) devices that efficiently promote deep muscle contraction have not been characterized. The purpose of this study was to clarify using [18F]-fluorodeoxyglucose positron emission tomography (FDG-PET) whether interference with low-frequency EMS can promote muscle metabolism in deep.

**Method:**

A total of 16 healthy males were randomly assigned to the EMS group (n = 8, age = 29.8 ± 4.1 years) or the control group (n = 8, age = 26.1 ± 5.0 years). Individuals in the EMS group received interference low-frequency EMS for 20 min each day over a span of three consecutive days as part of the pretest phase. On the measurement day, an EMS was conducted for 10 min, followed by an injection of FDG and then another EMS for an additional 10 min. The control group remained in a seated position for 10 min, after which FDG was administered intravenously. Images from PET-computed tomography were acquired in each group 60 min after the injection of FDG. Regions of interest were delineated in each muscle of the lower extremities. We analyzed the metabolism of each skeletal muscle sample by employing a standardized uptake value.

**Results:**

Compared with the control group, the EMS group showed increased glucose metabolism in both superficial and deep muscles of the trunk, pelvis, and lower extremities, as measured by FDG uptake.

**Conclusion:**

This exploratory study suggests that low-frequency interference EMS may increase glucose metabolism in deep and superficial muscles of the trunk, pelvis, and lower extremities.

**Supplementary Information:**

The online version contains supplementary material available at 10.1007/s00421-025-05847-6.

## Introduction

Japan's population is aging more swiftly than that of any other nation. Kojima et al. reported that the pooled prevalence of frailty was 1.9%, 3.8%, 10.0%, 20.4%, and 35.1% for individuals aged 65–69, 70–74, 75–79, 80–84, and ≥ 85 years, respectively (Kojima et al. 2017). Muscles that tend to deteriorate with age include the quadriceps and gluteus maximus muscles, which are involved in standing and walking, and the transversus abdominis and iliopsoas muscles, which are involved in maintaining posture, have also been reported to progressively atrophy with age (Chi et al. 2015). These muscles are also considered important in preventing falls, and intervention in deep muscles is an important issue in preventing falls that result in the need for nursing care.

In the rehabilitation field, interventions for deep muscles are also called core muscle training, and exercises that involve slow movements and maintaining a certain posture, rather than vigorous movements, are recommended (Akuthota and Nadler 2004). However, these exercise methods often have a large compensatory effect on superficial muscles in elderly individuals, making it difficult to fully activate deep muscles. Electrical muscle stimulation (EMS), which can elicit passive muscle contraction, serves as a valuable intervention tool for patients who struggle with effective voluntary muscle contraction. It is well-established that EMS can enhance muscle performance and increase muscle thickness (3–5). However, it has been confirmed that low-frequency EMS used for muscle strengthening, etc., has a strong stimulating effect on superficial muscles but only a small effect of stimulation on deep muscles (Numata et al. 2016). Interference wave stimulation is reported to be effective for deep muscle stimulation; however, it is frequently utilized for pain relief rather than muscle strengthening (Fuentes et al. 2010). The pulse width is crucial for eliciting muscle contraction through EMS, and the medium frequency of approximately 4000 Hz employed in interference waves is inadequate for inducing muscle contraction because of its excessively short pulse width (Lagerquist and Collins 2010). In this study, we focused on interference low-frequency devices that use a low frequency of approximately 400 Hz to increase the pulse width. With low-frequency interference, the pulse width can be extended to approximately 100 µsec, thereby facilitating muscle contraction. However, there have been no reports to date examining the intervention effects of interference with low-frequency waves in deep muscles.

Interferential low-frequency EMS uses two medium-frequency currents (e.g., 400 Hz and 420 Hz) that intersect in tissue to generate a low-frequency beat (20 Hz). Medium-frequency currents are known to reduce skin impedance, allowing for deeper current penetration (Goats 1990). Moreover, the interference pattern results in a pulse width of approximately 400 µs, which is longer than that of typical EMS devices and is more effective in depolarizing deeper motor neurons (Lagerquist and Collins 2010). The modulated beat frequency of 20 Hz is within the effective range for muscle activation and mimics voluntary contraction rhythms used in rehabilitation settings. Compared to conventional EMS or belt-type EMS systems that often use fixed electrode positions and low-frequency pulsed currents, our approach provides both enhanced comfort and increased depth of stimulation due to the combined effects of waveform, pulse width, and electrode configuration.

Electromyography and ultrasound have been employed as evaluative methods to assess muscle activity in various skeletal muscles. Nonetheless, these techniques are not applicable for assessing the deep musculature of the trunk, pelvis, and lower limbs. Consequently, we employed positron emission tomography (PET) to assess all skeletal muscles, including the deep muscles of the trunk, pelvis, and lower extremities. PET is a nuclear medicine modality that generates images of metabolic activities through the administration of a radioactive metabolic agent or its analog into the organism. [18F]-Fluorodeoxyglucose (FDG) features a structure where the radioactive isotope 18F replaces a hydroxyl (OH) group in the glucose molecule, allowing it to be absorbed by the body in a manner akin to that of glucose. This advantage has led to its frequent application in the local diagnosis of cancers within clinical settings. Nonetheless, it can also be used to examine glucose metabolism in the entirety of skeletal muscles throughout exercise (Nakase et al. 2013). Glucose metabolism assessed through FDG-PET is strongly correlated with muscle activity intensity, and its reliability as a measure of muscle activity has been validated (Pappas et al. 2001; Kemppainen et al. 2002).

Given the lack of definitive evidence of interferential low-frequency EMS on deep muscle activation, this study was designed as an exploratory investigation. Our aim was to determine whether this form of EMS increases glucose metabolism in deep skeletal muscles of the trunk, pelvis, and lower extremities, as measured by FDG-PET. Although our long-term goal is to inform future investigations for elderly or clinical populations, this foundational study was conducted in healthy young males to establish baseline effects under controlled conditions.

## Materials and methods

### Subjects

A total of 16 healthy males were randomly assigned to the EMS group (age = 29.8 ± 4.1 years, height = 173.0 ± 4.7 cm, weight = 63.9 ± 7.4 kg) or the control group (age = 26.1 ± 5.0 years, height = 170.2 ± 5.9 cm, weight = 62.8 ± 9.0 kg). All participants were considered healthy after a review of their medical history. The exclusion criteria were as follows: (1) diabetes mellitus and (2) allergy to drugs ([^18^F] FDG). All procedures were performed in accordance with the Declaration of Helsinki and were approved by the Kanazawa University Committee on Ethics in Research (approval number: 2024–001(6145)). This study was registered at jRCT (jRCT1041240059).

### Protocol

**Fig. 1:**
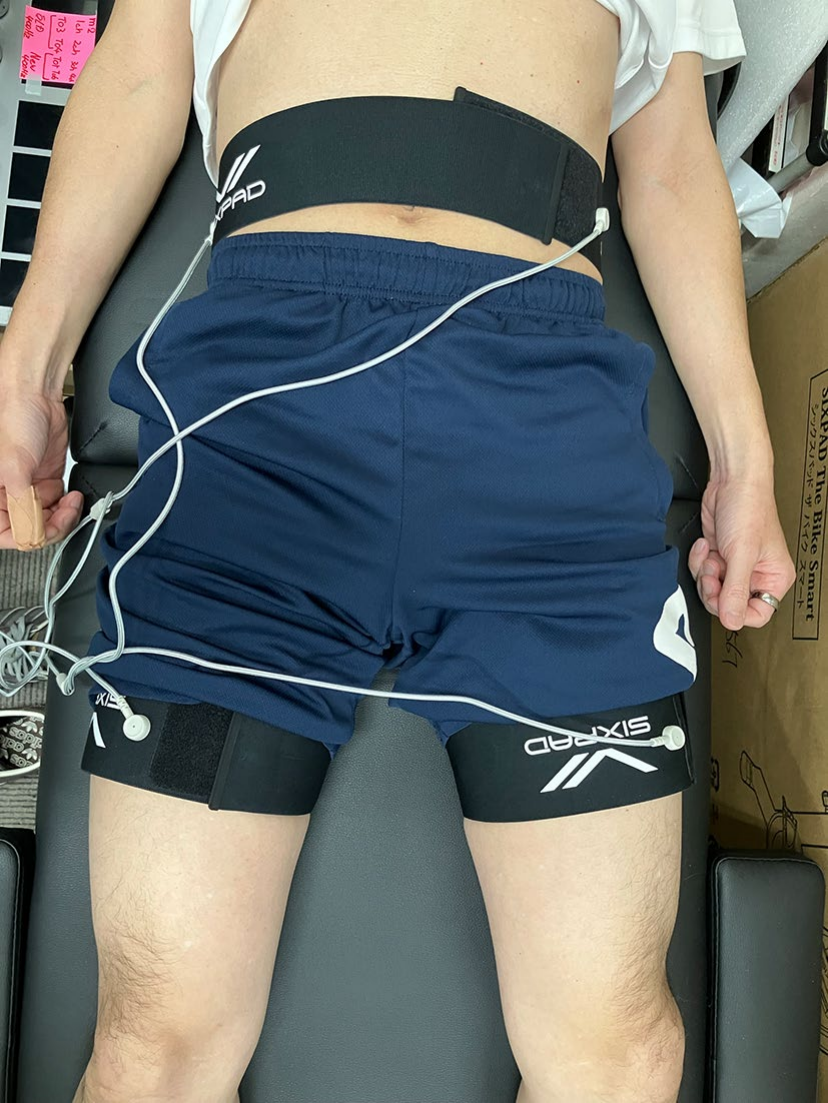
Placement of electrodes

The EMS group received PET-computed tomography (CT) scanning following interferential low-frequency EMS with the EMS device (SIXPAD Medical Pro, MTG Ltd, Nagoya, Japan), whereas the control group had PET-CT scanning after a period of rest. In the EMS group, participants wore electrode bands at the waist and proximal femurs (Fig. 1). The electrode bands were moistened with water, and a fixed-frequency current of 400 Hz, along with a modulated frequency of 420 Hz, was applied, leading to an effective frequency of 20 Hz and a current intensity varying from 0.7 to 28.0 mA. The output intensity was established at the permissible maximum level. Given that participants had prior experience with the EMS, the aim was to achieve the highest tolerable maximum output on the measurement day. Therefore, EMS was conducted for 20 min daily over three consecutive days as a preparatory exercise. On the test day, the tracer was administered intravenously following 10 min of EMS, and PET‒CT imaging was carried out for an additional 10 min (Fig. 1) (Numata et al. 2016). The participants in the control group were instructed to maintain a seated posture for 10 min. The tracer was subsequently administered intravenously, and PET‒CT imaging was subsequently performed. PET‒CT images were obtained from each group 60 min after the FDG injection; [18F] FDG 37 MBq (a glucose derivative labeled with the radioactive isotope 18F) was used as the tracer (Fujimoto et al. 1996; Nakase et al. 2013).

Prior to PET‒CT imaging, all participants were directed to refrain from food intake for a duration of 6 h before the examination. Additionally, they were advised to limit physical activity, allowing only for routine daily tasks, for the day leading up to the test. The blood glucose level of each participant was evaluated to confirm that it remained within the normal range. A Discovery PET/CT 690 scanner (GE Healthcare, situated in Milwaukee, WI, USA) was used, with an imaging duration of approximately 40 min (Nakase et al. 2013). The radiation exposure levels linked to the intravenous injection of FDG and the CT scanner used in this study were approximately 1 mSv and 4 mSv, respectively.

### PET analysis

Regions of interest (ROIs) were delineated according to the anatomical nomenclature of muscles discernible on a plain CT image. This included the rectus abdominis, external oblique, internal oblique, transversus abdominis, psoas major, iliac, gluteus maximus, gluteus medius, gluteus minimus, sartorius, quadriceps femoris, biceps femoris, semitendinosus, semimembranosus, and adductor muscle complex. The skeletal muscles within the three distinct regions (trunk, pelvis, and thigh) were systematically identified in the cross-sectional area, which was designed to facilitate identification. The complete cross-sectional area of each skeletal muscle was defined as the zone of interest on both the right and left sides. The cross-section at the superior margin of the fourth lumbar vertebra was employed for the trunk; the section at the superior margin of the acetabulum was allocated for the pelvis; and the section at the midpoint of the inferior margin of the femoral lesser trochanter and the femoral condyle was utilized for the lower leg. CT scans were employed to outline each muscle, and the assessment of FDG uptake was performed by comparing these CT images with the PET‒CT images. The assessment of glucose metabolism in each ROI was performed using the standardized uptake value (SUV), which is a semiquantitative metric for comparing glucose metabolism among muscles (10). ROIs were manually delineated using OsiriX MD (Pixmeo SARL, Geneva, Switzerland), a commercially available DICOM viewer, based on CT-defined anatomical landmarks such as muscle fascia and bone margins. ROI delineation was guided by a predefined protocol that utilized identifiable anatomical landmarks on axial CT slices. Consistency was further ensured by using standardized axial slice levels (e.g., L4, acetabular, mid-femoral planes) and applying uniform DICOM window settings within OsiriX MD. PET–CT image alignment was carefully verified prior to segmentation. Although only one experienced physician performed the segmentation, these standardized procedures were implemented to enhance reproducibility and methodological rigor. All ROIs were drawn by a single board-certified physician with extensive experience in musculoskeletal PET–CT interpretation, who was blinded to group allocation. Although the use of a single experienced rater was intended to minimize segmentation variability, inter- and intra-rater reliability was not formally assessed. Future studies should consider involving multiple experienced raters and formal reliability testing to enhance reproducibility. The SUV represents the ratio of the local concentration, which is predicated on the equivalency of the tissue concentration, and it is assumed that the radioactivity is normalized to 1 when the injected radioactive isotope is uniformly disseminated and retained throughout the body. The SUV is calculated using the following formula: SUV = (counts in the ROI [cps/mL] × the inverse of the calibration constant [Bq/cps] ÷ (the injected dose [Bq]) ÷ body weight [g]). The average SUV was calculated using the following formula:

$$mean SUV= \frac{\left(left mean SUV\times left muscle area+right mean SUV\times right muscle area\right)}{left muscle area+right muscle area}$$

A comparative examination of skeletal muscle metabolic activity between the two groups was performed using the mean SUV.

### Statistical analysis

All the statistical analyses were performed using Stata version 17 (Stata Corp LLC, Texas, USA). The sample size was established in accordance with our prior investigation of muscle activity utilizing FDG-PET following EMS (Numata et al. 2016). Significant differences in muscle activity were observed in the vastus lateralis muscle, with measurements of 2.09 ± 0.75 in the EMS group compared with 0.54 ± 0.10 in the control group. According to these values, the required number of samples was determined with a significance level of 0.05 and a power of 80%, leading to the conclusion that 5 cases per group would be essential. In light of potential unforeseen withdrawals, a total of 16 patients were enrolled in this study, with 8 patients allocated to each group.

All the data are expressed as the means ± standard deviations. A comparison of the SUVs across all the ROIs between the groups was conducted using an unpaired t test. The significance threshold was established at p < 0.05. Given the multiple comparisons across different muscle ROIs, we applied the Benjamini–Hochberg false discovery rate (FDR) correction to control for the risk of type I error. All reported *p*-values were adjusted using this method, and results with adjusted *p* < 0.05 were considered statistically significant. The effect size was determined using Cohen’s d and was calculated via the following equation:

$$d=\frac{\left|{\overline{x} }_{1}-{\overline{x} }_{2}\right|}{{S}_{c}}$$

$${S}_{c}= \sqrt{\frac{{n}_{1}{S}_{1}+{n}_{2}{S}_{2}}{{n}_{1}+{n}_{2}}}$$

where ${\overline{x} }_{1}$ and ${\overline{x} }_{2}$ are the mean SUV data of the control and EMS groups, *n*_*1*_ and *n*_*2*_ are the sample sizes of the control and EMS groups, and *S*_*1*_ and *S*_*2*_ are the sample variances of the control and EMS groups. Cohen’s d values were reported unadjusted, as they represent standardized differences in group means that are independent of statistical significance and are not affected by multiple comparison corrections.

## Results

There were no significant differences in individual characteristics, such as age (*p* = 0.1350), height (*p* = 0.3102), weight (*p* = 0.8027), or body mass index (*p* = 0.7480), between the groups.

**Fig. 2:**
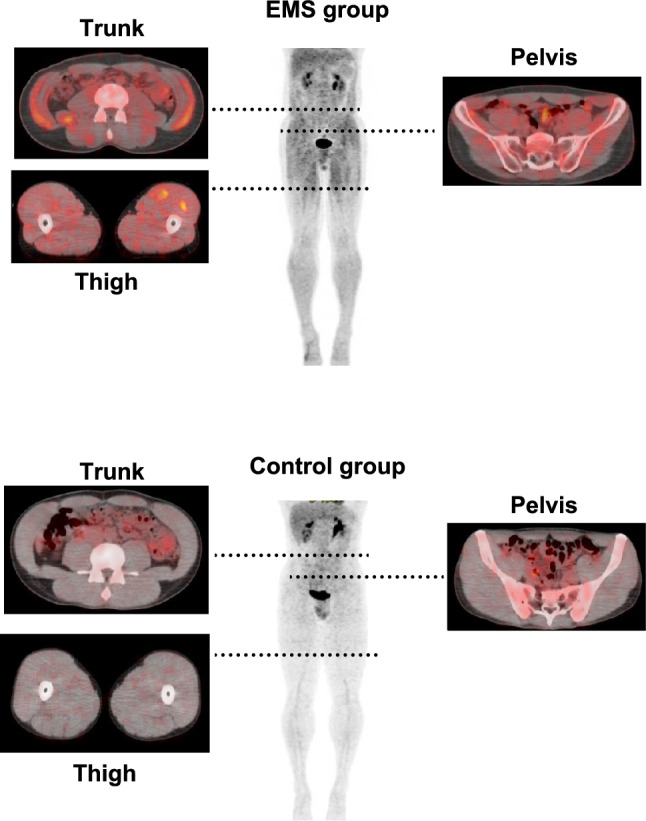
Representative whole-body PET images. The EMS group (upper panel) shows increased FDG uptake compared to the control group (lower panel), indicating elevated glucose metabolism in both superficial and deep muscles, including the iliopsoas and transversus abdominis

Figure 2 shows representative whole-body PET images of the EMS group (Fig. 2 upper panel) and control group (Fig. 2 lower panel). Compared with the control group, the EMS group presented FDG accumulation in each muscle. To evaluate differences in muscle activity, we compared the mean standardized uptake values (SUVs) of each muscle between groups. Given the number of comparisons (15 muscles), we applied the Benjamini–Hochberg false discovery rate (FDR) correction to control for Type I error. All 15 muscles showed statistically significant increases in SUV in the EMS group compared to the control group after FDR correction (adjusted *p* < 0.05). Moreover, the iliopsoas muscle exhibited a medium effect size, whereas the other muscles exhibited large effect sizes, indicating the substantial impact of EMS on deep and superficial muscle activation (Table 1).

**Table 1:** Mean SUV in the control and EMS group (FDR-corrected)

Muscle	Control SUV (mean ± SD)	EMS SUV (mean ± SD)	p-value	p-value (FDR adj)	95% CI	Cohen's d
Rectus abdominis	0.51 ± 0.06	0.80 ± 0.23	0.0038	0.0041	0.11–0.47	1.85
External oblique	0.48 ± 0.05	1.24 ± 0.18	0.0010	0.0014	0.61–0.90	5.99
Internal oblique	0.55 ± 0.06	1.17 ± 0.33	0.0002	0.0010	0.36–0.87	2.77
Transversus abdominis	0.58 ± 0.07	0.95 ± 0.23	0.0007	0.0013	0.19 to 0.55	2.33
Psoas major	0.76 ± 0.11	1.24 ± 0.24	0.0002	0.0008	0.28–0.68	0.73
Iliac	0.70 ± 0.07	1.52 ± 0.20	0.0001	0.0015	0.66–0.99	2.41
Gluteus minimus	0.68 ± 0.06	1.47 ± 0.53	0.0008	0.0013	0.39 to 1.20	2.66
Gluteus medius	0.61 ± 0.07	1.36 ± 0.46	0.0004	0.0009	0.40–1.10	3.36
Gluteus maximus	0.50 ± 0.07	0.80 ± 0.25	0.0051	0.0051	0.11–0.50	1.69
Sartorius	0.49 ± 0.04	1.36 ± 0.39	0.0001	0.0007	0.56–1.16	1.51
Quadriceps femoris	0.49 ± 0.09	0.72 ± 0.10	0.0003	0.0007	0.13 to 0.33	2.57
Biceps femoris	0.45 ± 0.07	0.75 ± 0.36	0.0010	0.0013	0.14–0.45	2.23
Semitendinosus	0.44 ± 0.06	0.61 ± 0.07	0.0002	0.0006	0.01 to 0.24	2.72
Semimembranosus	0.43 ± 0.06	0.54 ± 0.06	0.0032	0.0037	0.04–0.17	1.90
Adductor complex	0.51 ± 0.04	0.71 ± 0.13	0.0008	0.0012	0.10–0.30	2.30

*SUV* standardized uptake value, *EMS* electrical muscle stimulation, *FDR* false discovery rate

## Discussion

The purpose of this study was to demonstrate the influence of interference with low-frequency EMS on deep muscles. Given the lack of a predefined hypothesis regarding specific muscle groups, the present study was designed as an exploratory investigation. Our notable finding was that interference with low-frequency EMS led to increased glucose metabolism in both superficial and deep muscles, as visualized through FDG-PET imaging. This metabolic response suggests that such stimulation protocols may have the potential to activate musculature throughout the trunk, pelvis, and lower extremities. However, given that FDG uptake reflects glucose utilization rather than direct contractile activity, the functional significance of this activation requires further investigation. While previous studies have reported EMS-induced muscle activity using PET or EMG, the present study adds to the literature by demonstrating that low-frequency interference wave EMS is associated with increased glucose uptake in deep muscles such as the iliopsoas and transversus abdominis. The integration of FDG-PET with this specific stimulation protocol provides a novel methodological contribution, although complementary physiological markers (e.g., EMG or force output) will be necessary in future studies to confirm functionally meaningful muscle recruitment.

It is widely known that EMS can improve muscle performance and muscle thickness in several age groups (young to elderly) (Bélanger et al. 2000; Maffiuletti 2010; Nishikawa et al. 2019, 2021). There are several EMS parameters, which can vary widely depending on the goals of the intervention task. In general, most clinical regimens use 20–50 Hz patterns for optimal results (Baker et al. 1988; de Kroon et al. 2005). To avoid fatigue or discomfort, constant low-frequency stimulation is typically used in the clinical field. Our research group reported that low-frequency EMS (20 Hz), which is generally used for muscle strengthening purposes, stimulates superficial muscles, but no apparent increase in muscle activity was observed in deep muscle using FDG-PET (Numata et al. 2016).

Therefore, low-frequency EMS was considered unsuitable for deep muscle stimulation. This study sought to target deep muscles using interferential current. Interferential current is a prevalent electrotherapeutic technique employed for pain management (Fuentes et al. 2010). In general, interferential current is the application of alternating medium-frequency current (4000 Hz) amplitudes modulated at low frequencies (0–250 Hz) (Goats 1990). A claimed advantage of interferential current over lower-frequency current is its capacity to diminish the impedance offered by the skin (Ward 1980). Another proposed benefit of interferential current is its capacity to produce an amplitude-modulated frequency parameter, which is a low-frequency current created deep within the treatment region (Goats 1990). Thus, interferential current is used in the rehabilitation field to stimulate deep muscles, mostly for pain relief (Fuentes et al. 2010). There have been few studies aimed at increasing muscle strength, and no reports have assessed muscle activity in deep muscles in detail. We previously confirmed muscle contraction by ultrasound echo using interference waves from medium frequencies (4000 Hz and 4020 Hz) but could not confirm muscle contraction in deep muscles (Supplemental Fig. 1). We attributed this factor to the pulse width of the EMS. Duty cycles of 1:2 or 1:3 are commonly used in clinical practice (Doucet et al. 2012), with shorter pulse widths for medium- or higher-frequency currents. It has been reported that longer pulse widths can stimulate stronger contractions (Lagerquist and Collins 2010), and increasing the pulse width to stimulate muscle contraction is considered necessary. Based on these considerations, we selected a pulse width of 400 μs in this study to enhance the likelihood of muscle contraction, particularly in deep muscle layers. The results of this study demonstrated that stimulation with interferential current and wide pulses was associated with increased FDG uptake in several deep muscles, indicating elevated glucose metabolism. However, this metabolic response alone does not confirm preferential or functionally meaningful activation, as FDG-PET measures glucose uptake and cannot differentiate between contractile activity and passive metabolic processes.

While interference EMS produces a low-frequency modulation envelope (e.g., 20 Hz) through the intersection of two medium-frequency currents, the physiological equivalence of this modulation to conventional low-frequency stimulation remains to be fully established. Medium-frequency currents are known to reduce skin impedance, allowing for deeper tissue penetration (Goats 1990; Ward and Robertson 1998). Furthermore, the interference pattern results in an extended effective pulse width (~ 400 µs), which is more likely to activate larger, deeper motor units (Lagerquist and Collins 2010). However, the assumption that the 20 Hz beat frequency behaves physiologically like traditional low-frequency EMS has not been fully validated in skeletal muscle. We acknowledge this as a limitation of the current study, and future electrophysiological investigations (e.g., EMG, nerve excitability tests) are necessary to confirm the specific neuromuscular recruitment patterns induced by low-frequency interference EMS.

This study has several limitations. First, all participants were healthy young males. Therefore, the results may not be generalizable to females or older individuals. Previous studies have shown sex- and age-related differences in muscle fiber composition and metabolic responses to EMS (Simoneau et al. 1985; Lexell and Taylor 1991; Miller et al. 1993; Miyamoto et al. 2015; Bougea et al. 2016). Future studies should include more diverse populations to improve external validity. Second, the study utilized a between-subjects design with a small sample size (n = 8 per group), which may have introduced interindividual variability and limited statistical power. Although a priori power analysis was performed, it did not account for the multiple comparisons across muscle ROIs. To mitigate this, we applied the Benjamini–Hochberg FDR correction. Nevertheless, larger-scale studies employing within-subject or paired-limb designs are needed to enhance precision and control for baseline differences. Third, the study was exploratory in nature and did not include a priori hypotheses about which muscles would be most responsive to EMS. Future investigations should employ hypothesis-driven designs with predefined regions of interest to validate and extend the current findings. Fourth, ROI delineation was performed manually by a single experienced physician. Although this minimized variability, inter- and intra-rater reliability was not assessed. To improve segmentation consistency, ROI delineation followed a predefined protocol using identifiable anatomical landmarks on CT, standardized axial slice levels (e.g., L4, acetabular, and mid-femoral planes), and uniform DICOM viewing settings in OsiriX MD. PET–CT image alignment was also carefully verified before segmentation. Future studies should incorporate multiple expert raters and reliability testing for segmentation consistency. Fifth, FDG-PET provides valuable metabolic information but cannot directly measure contractile function. Increased glucose uptake may reflect passive uptake, perfusion changes, or metabolic inefficiency rather than functional activation. Moreover, SUV measures do not differentiate between active and passive processes. Future research should include physiological correlates such as EMG, torque, or muscle force to verify neuromuscular recruitment. Sixth, FDG uptake was measured at a single time point and limited to specific anatomical slices, which may not capture full muscle activation or longer-term physiological responses. Furthermore, we did not assess delayed-onset muscle soreness (DOMS), strength changes, or recovery status. These limitations preclude conclusions about training effects or adaptation. Temporal tracking and functional assessments should be incorporated in future studies. Seventh, although the study was motivated in part by potential applications in aging populations and fall prevention, the current findings serve only as foundational data. Application to clinical or elderly populations will require separate validation.

## Conclusion

This exploratory study suggests that low-frequency interference EMS may induce increased glucose metabolism in muscles of the trunk, pelvis, and lower extremities, including deep muscles such as the iliopsoas and transversus abdominis. While these findings provide foundational evidence for potential applications of this stimulation protocol, further studies are needed to confirm its functional effects and evaluate its efficacy in clinical or aging populations.

## Supplementary Information

Below is the link to the electronic supplementary material.

Supplementary file1 (TIF 795 KB)

## Data Availability

Data are available from the corresponding author upon reasonable request.
