# Supplementary figures and images for: Increased deep muscle activity with interference low-frequency electrical muscle stimulation: evaluation by positron emission tomography
Source: Eur J Appl Physiol. 2025 Jun 12;125(11):3377–84. doi: 10.1007/s00421-025-05847-6 (PMC12528247; doi:10.1007/s00421-025-05847-6)

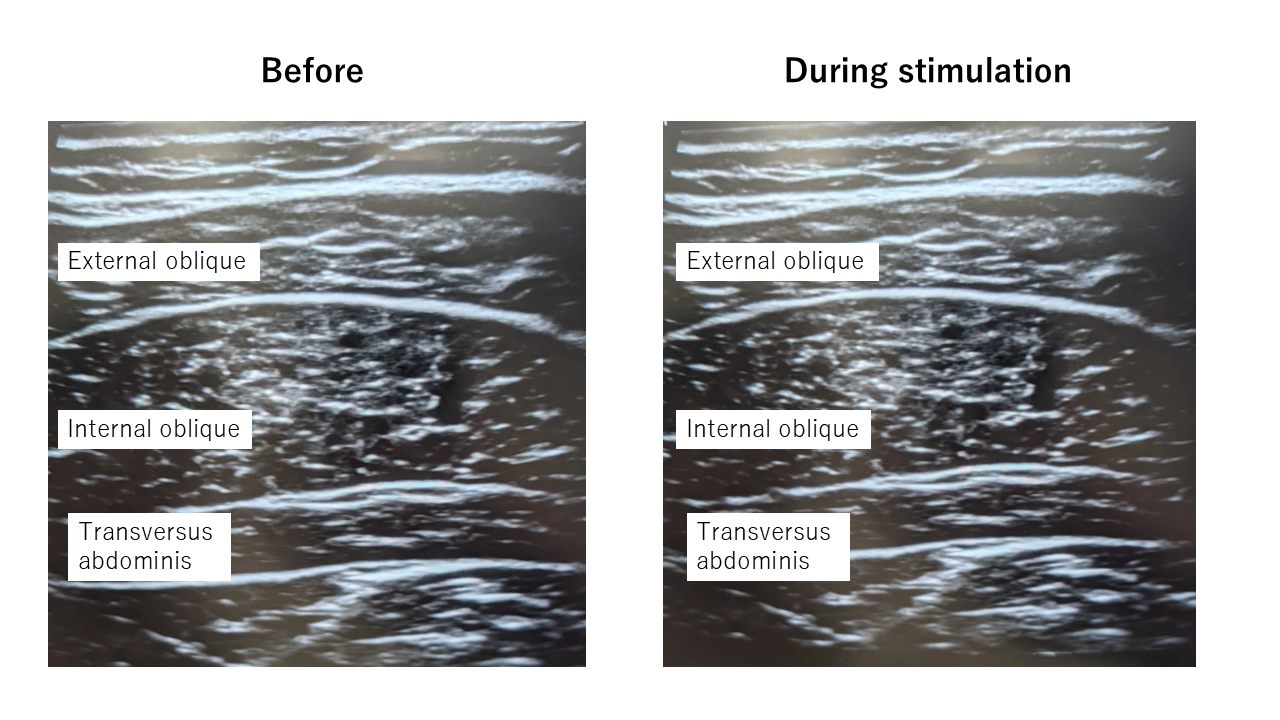

Supplement: Supplementary file 1 — Supplementary file1 (TIF 795 KB) [file 421_2025_5847_MOESM1_ESM.tif]
